# Polyostotic osteosarcoma associated with avian leukosis virus infection in a captive bare‐faced curassow (*Crax fasciolata*)

**DOI:** 10.1186/s12917-021-02794-0

**Published:** 2021-02-18

**Authors:** Jefferson Bruno Soares Oliveira, Ayisa Rodrigues de Oliveira, Daniel Oliveira dos Santos, Thaynara Parente de Carvalho, Larissa Giannini Alves Moreira, Herlandes Penha Tinoco, Carlyle Mendes Coelho, Hannah Luiza Gonsalves Coelho, Maria Clara de Paiva Zucherato, Sandra Yuliet Marín-Gómez, Camila Siqueira Costa, Nelson R. S. Martins, Renato Lima Santos

**Affiliations:** 1 Departamento de Clínica e Cirurgia Veterinária, Escola de Veterinária, Universidade Federal de Minas Gerais, Av. Presidente Antônio Carlos, 6627 – CEP 30161-970, Minas Gerais 31270-901 Belo Horizonte, Brazil; 2 Hospital Veterinário – Fundação de Parques Municipais e Zoobotânica de Belo Horizonte, 31365-450 Belo Horizonte, Minas Gerais Brazil

**Keywords:** Bone tumor, ALV, Cracidae, Wildlife, Retrovirus

## Abstract

**Background:**

Osteosarcoma is a malignant mesenchymal bone tumor. Although it is a common tumor in the appendicular skeleton of dogs and cats, it is rarely reported in birds. Retroviruses are usually associated with solid tumor development in different avian species.

**Case presentation:**

This report aims to describe a case of osteosarcoma associated with the avian leukosis virus in a captive bare-faced curassow (*Crax fasciolata*). A captive adult female bare-faced curassow presented with lameness, hyporexia, and a non-ulcerative and firm tumor in the right femur. The bird was euthanized due to the poor prognosis. Histopathology revealed an infiltrative mesenchymal neoplasm consisting of spindle cells with moderate cell pleomorphism, organized in bundles and interspersed by marked deposition of the osteoid matrix, which was compatible with osteosarcoma affecting both femur and tibiotarsus, with renal metastasis. Immunohistochemistry of the primary and metastatic tumor demonstrated vimentin expression by neoplastic cells. Samples of the neoplasm, bone marrow, and spleen were processed for PCR, which enabled the demonstration of proviral avian leukosis virus (ALV) DNA.

**Conclusions:**

To the best of our knowledge, this is the first report of an osteosarcoma in a bare-faced curassow with an unusual polyostotic manifestation and associated with ALV infection.

## Background

Osteosarcoma is a malignant bone tumor originated of pluripotent mesenchymal cells that commonly affects the appendicular and axial skeleton. Less frequently, this tumor may be primary of soft tissues when it is classified as an extra-skeletal form [1–3]. Among domestic animals, osteosarcoma affects mainly dogs and cats between 8 and 10 years of age, with poor prognosis, and a high metastatic rate [4–6]. In birds, osteosarcoma is uncommon and there are only a few cases reported in domestic and wild species [7–9], in which it may arise in the axial or, more commonly, in the appendicular skeleton [7, 8, 10, 11].

Certain infectious agents play an import role in oncogenesis. In domestic animals retroviruses are commonly associated with tumor manifestations in different species, including cattle, sheep, cats, and chickens [12–15]. In birds, the most important retroviruses associated with neoplasms are the Avian Leukosis Virus (ALV, *Alpharetrovirus*) and the Reticuloendotheliosis Virus (REV, *Gammaretrovirus*) [16–18]. Myelocytic or lymphoproliferative tumors and leukemia, as well as osteopetrosis, may occur in chickens infected with ALV [19, 20].

The bare-faced curassow (*Crax fasciolata*, Cracidae) is widely distributed in the Brazilian territory, and other Latin American countries. These birds are of great importance for the maintenance of forests due to their seed-based feeding, contributing to the recovery of deforestation areas [21]. According to the Institute Union for Conservation of Nature, this species is considered *vulnerable* to the risk of extinction [22]. One of the main causes that endanger the existence of this species is hunting, mainly observed in areas without environmental protection [23]. In Brazil, a health assessment of different Cracidae species, including *C. fasciolata* (n = 28) has demonstrated exposure to chicken pathogens [24]. The present report aims to describe a case of osteosarcoma associated with avian leukosis virus infection in a captive bare-faced curassow.

## Case presentation

**Fig. 1:**
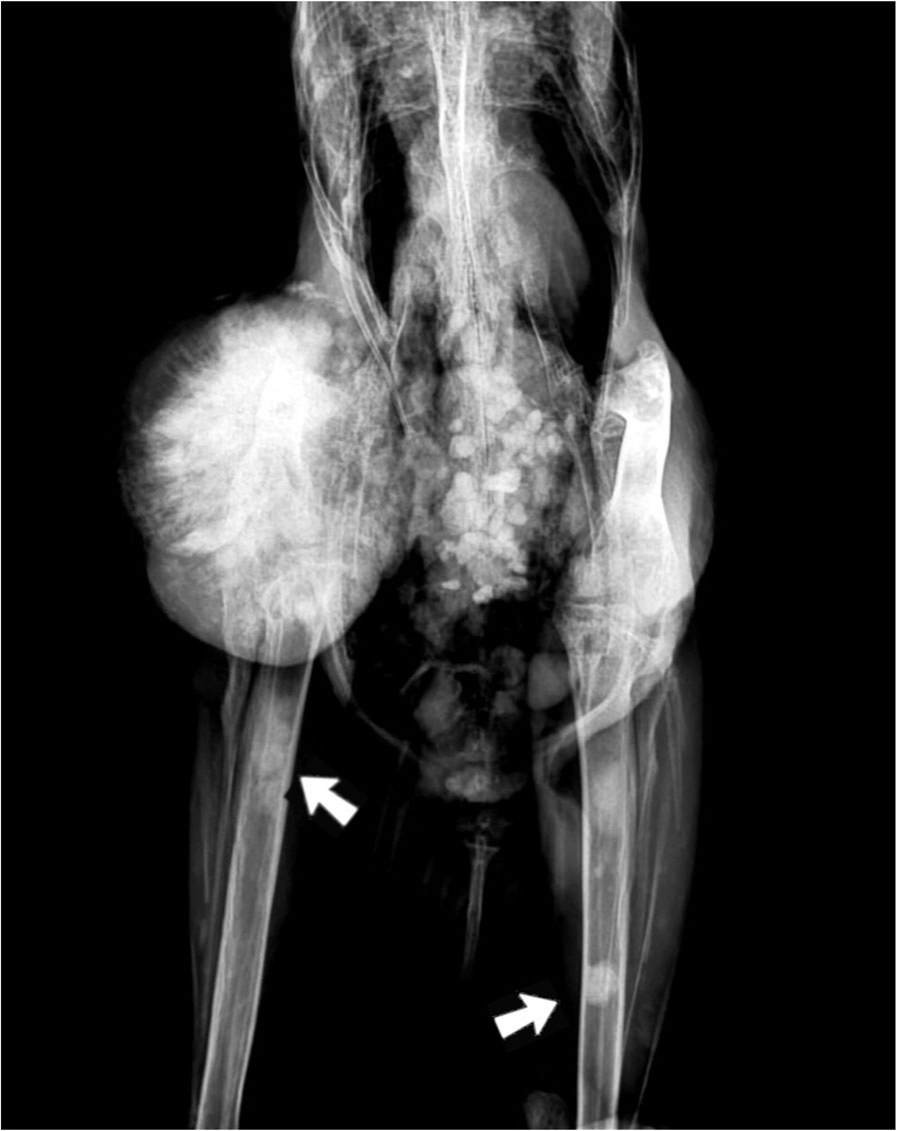
Radiological findings in an adult female bare-faced curassow (*Crax fasciolata*) with polyostotic osteosarcoma. Increased volume of the right femur associated with a focal area of extensive radiopacity. Within the medullary canal there are focal areas of increased radiopacity (arrows)

A captive adult female bare-faced curassow (*Crax fasciolata*) housed at the Belo Horizonte Zoological Garden presented lameness and hyporexia, and was admitted to the veterinary hospital. Physical examination revealed a firm non-ulcerative tumor in the right femur with approximately 10 cm in diameter. Radiographic examination showed a radiopaque proliferation, with irregular edges and bone lysis at the diaphysis of the right femur, which extended to the tibiotarsal joint. Multiple radiopaque areas were also observed in the medullary canal of the right tibiotarsus, left femur, and left tibiotarsus (Fig. 1). Due to the poor prognosis, the bird was euthanized performing anesthesia induction with inhalation of 5 % isoflurane, followed by intravenous propofol (0.04 mg/kg). Once under deep anesthesia the animal received 100 mg/kg of potassium chloride. Necropsy was performed immediately.

**Fig. 2:**
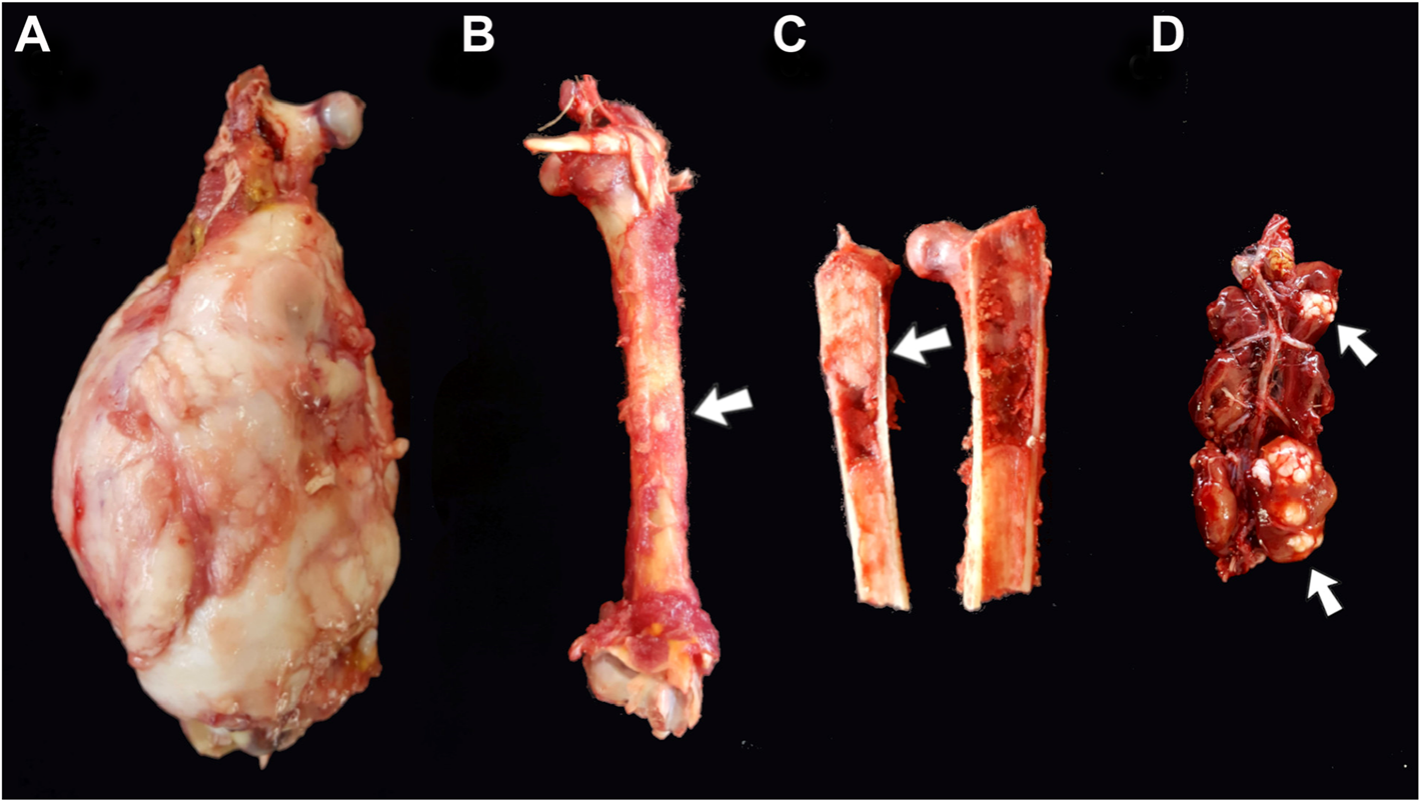
Gross findings in an adult female bare-faced curassow (*Crax fasciolata*) with polyostotic osteosarcoma. **a** nodular growth in the right femur involving the diaphysis and distal metaphysis. **b** Nodule in the contralateral femur, which was whitish solid color and hard consistency (arrow). **c** Neoplastic proliferation in the left femur, filling a focal area of the medullary cavity extending to the cortical region (arrow). **d** Kidney with multifocal nodular areas of neoplastic proliferation similar to those found in the pelvic limbs

The bird was cachectic with marked hypotrophy of the thoracic muscles and a cutaneous abrasion on the keel skin. Grossly, the right femur had a whitish firm mass of 10 × 10 × 7 cm completely involving the diaphysis and distal metaphysis (Fig. 2a). On cut surface, the neoplasm was solid, white, firm, and infiltrated the medullary space. On the surface of the left femur there was a nodule with 0.2 cm of diameter with the same gross appearance as observed in the neoplasm of the right femur (Fig. 2b). In the medullary space of both femurs and tibiotarsus there were multiple areas with neoplastic osseous proliferation (Fig. 2 c). Additionally, the right kidney had two nodular irregular whitish firm areas, one with 0.5 cm of diameter and the other with 2.5 × 1.5 × 1.0 cm (Fig. 2d). Samples of the bone neoplasm and bone marrow, lungs, liver, heart, kidney, and brain were collected, fixed in 10 % buffered formalin and routinely processed for paraffin embedding and histopathology. Samples of the neoplasm and bones were decalcified in formic acid prior to further processing. Four-µm-thick sections were stained with hematoxylin and eosin (HE), Masson’s trichrome, alcian blue, and picrosirius red.

**Fig. 3:**
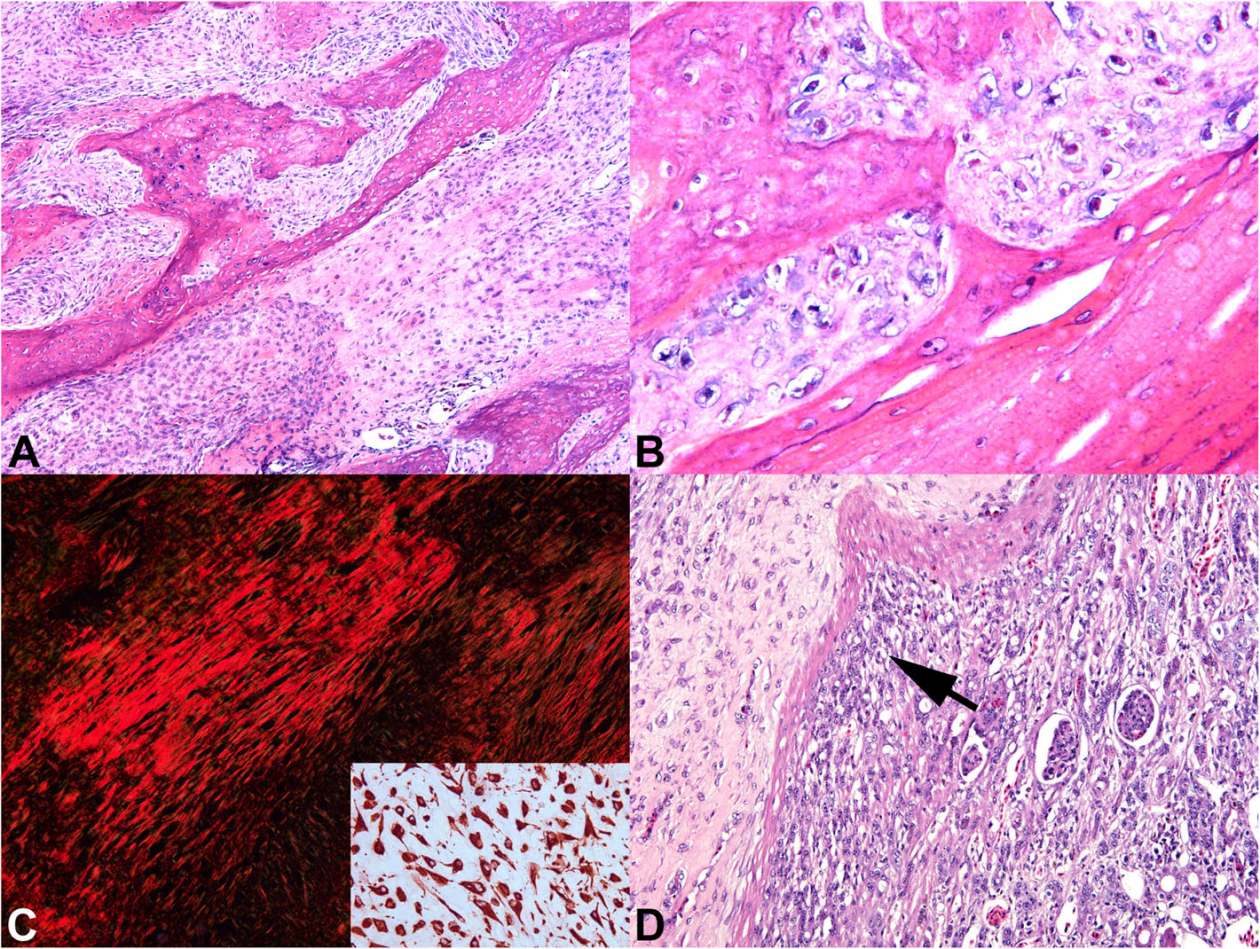
Microscopic findings in an adult female bare-faced curassow (*Crax fasciolata*) with polyostotic osteosarcoma. **a** The neoplasm exhibited infiltrative growth with cells organized in bundles interspersed by marked production of osteoid matrix and areas of mineralization. HE, 200X. **b** Cytologic features of malignancy characterized by a high nucleus:cytoplasm ratio, coarse aspect chromatin with evidence of nucleoli, being possible to observe binucleated cells. HE, 400X. **c** Evidence of type I collagen deposition synthesized by neoplastic cells. Picrosirius red under polarized light, 200X; Inset; strong vimentin immunostaining in neoplastic cells. **d** Metastatic neoplastic cell proliferation in the right kidney by compressing the tubes, altering the tissue architecture (arrow). HE, 200X

Microscopically, the tumor of the right femur was characterized by an infiltrative, poorly demarcated and non-encapsulated mesenchymal neoplastic proliferation that filled the entire space of the bone marrow and projected internally and externally from the cortex, which was partially preserved. The neoplasm was constituted on the periphery by spindle cells with moderate cellular pleomorphism, organized in bundles, presenting scarce basophilic cytoplasm, sometimes stellar, and interspersed by intense deposition of homogeneous eosinophilic matrix compatible with osteoid (Fig. 3a). These cells also exhibited a high nucleus: cytoplasm ratio, large and vacuolized nuclei with a predominance of central and very evident nucleolus, moderate anisocariosis, and nuclear pleomorphism (Fig. 3b). There were 20 mitosis figures per ten high power microscopic fields and rare binucleated cells. In the central region there was chondroid and osteoid differentiation. The left femur had a similar mesenchymal neoplastic tissue replacing the bone marrow and infiltrating the cortex, with nodules projecting on the periosteal surface. There were foci of chondroid and osteoid differentiation with marked deposition of a homogeneous eosinophilic matrix compatible with osteoid in all sections.

The right kidney had an expansive and encapsulated neoplastic proliferation with morphologic features similar to that of the neoplasm of the femur (Fig. 3d). Neoplastic cells had a high nucleus:cytoplasm ratio, with large and vacuolated nuclei with a predominance of central and prominent nucleolus, moderate anisokariosis and moderate nuclear pleomorphism. There were 25 mitotic figures per ten high power microscopic fields. There were foci of chondroid and osteoid differentiation with marked deposition of extracellular osteoid matrix.

Special stains including Masson trichrome, alcian blue and picrosirius red, evidenced type I collagen deposition synthesized by neoplastic cells, characteristic of osteoid matrix (Fig. 3 c), and allowed better recognition of areas with chondroid differentiation, and mineral deposition.

Immunohistochemistry of the primary bone tumor and the metastasis in the kidney was performed using an anti-vimentin mouse monoclonal antibody (RV202; Santa Cruz Biotechnology) at 1:100 dilution. Vimentin was consistently expressed by neoplastic cells (Fig. 3 c). Altogether, these findings were compatible with polyostotic osteosarcoma, due to multiple proliferation sites of the neoplasia in the bones and bone marrow, with renal metastasis.

Additionally, fragments of the neoplasm, bone marrow, and spleen were collected at necropsy and stored at -20°C for further evaluation. DNA extraction was performed using a chaotropic agent (sodium iodide-NaI) [25] and adsorption to silica as described by [26]. DNA samples were stored at -20°C. The quantity and purity of the DNA were determined by optical density in a NanoVue spectrophotometer (GE, Healthcare, UK).

The primers were designed using NCBI’s PrimerBlast to amplify the region of the polyprotein encoded by the gag gene (ALVG1 forward 5’-GGTCAGGACCAAGGGCTTAC–3’ and ALVG1 reverse 5’-GGGCACTGCGCTTGATAATG–3’). The amplification reaction was performed with 300 ng of template DNA, 1X PCR buffer (100 mM Tris-HCl pH8.4, 500 mM KCl), 0.4 nM of dNTP (containing dATP, dCTP, dGTP and dTTP, with a final concentration of 10 mM), 2 mM of MgCl_2_, 15 uM of each primer, 1.5 UI of Taq Polymerase (Taq DNA Polymerase, Phoneutria, Brazil), and Milli-Q Water 18.2 MΩ for a final volume of 25 uL. Reactions (40 cycles) were optimal when denatured at 95ºC for 1 minute (5 min at 94ºC was used for the first cycle), annealed at 50 C for 1 minute, and extended at 72ºC for 1 minute, and a final extension at 72ºc for 5 minutes in a thermocycler (Nyx Technik. Amplitronyx 4). PCR products (777 bp) were analyzed by agarose gel electrophoresis (1.5 %) followed by sequencing (Sanger dideoxynucleotide method). As negative controls, SPF chicken tissue DNA template and milliQ water replacing the template DNA were used. A positive control sample from a chicken diagnosed with avian leukosis (osteopetrosis) was included.

**Fig. 4:**
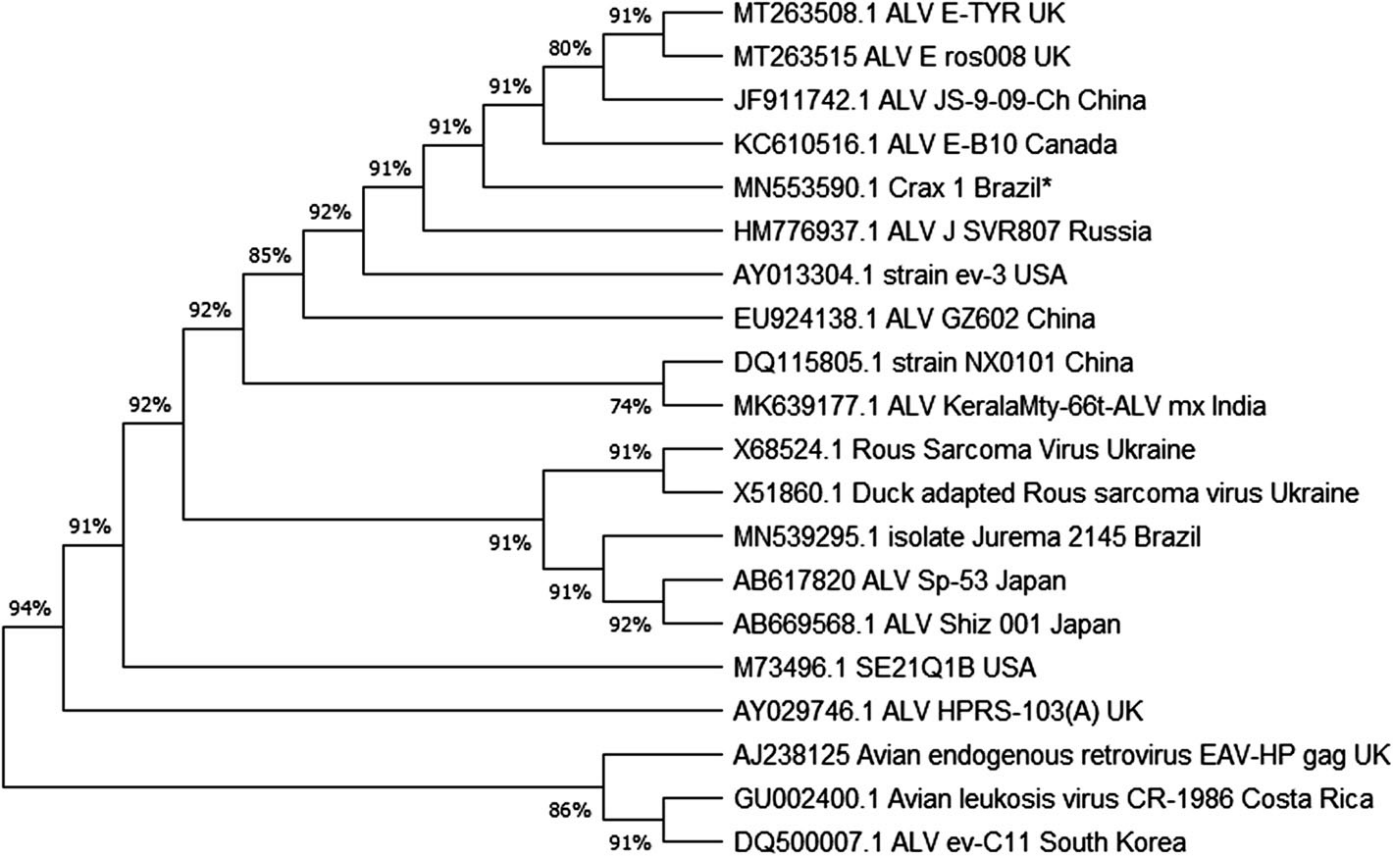
Evolutionary relationships of Avian Leukosis Virus strain Crax 1. Evolutionary relationships by maximum likelihood method of Avian Leukosis Virus strain Crax 1 detected in an adult female bare-faced curassow (*Crax fasciolata*) with osteosarcoma (asterisk) (accession number MN553590.1) were noted to ALV JS-9-09 (JF911742.1), described in 2009 in China, ALV-E B10 (KC610516.1) in 2013 in Canada, ALV-E TYR (MT263508.1) and ros008 (MT263515) both of 2020 in the United Kingdom, as these were grouped together in 91 % of the replicate trees, with a potential common ancestral to Crax 1 and these strains. All compared strains were found in chickens (*Gallus gallus domesticus*), described geographically worldwide, as are the chickens, and chronologically described from 2009 to 2020. At the farthest branches, strains ev-C11 (DQ500007) of South Korea was described in 2008, CR-1986 (GU002400) in 1986 in Costa Rica, and EAV-HP (AJ238125) in 2005 in the UK, seemed to be related to a common ancestral to all strains in the tree, including Crax 1, but the phylogenetic distance could not be associated to a chronological distance

The product sequences obtained had high identity with previously published avian lymphoid leukosis *gag* gene sequences (Fig. 4). The closest evolutionary relationships of Crax 1 (accession number MN553590.1) were detected with ALV JS-9-09 (JF911742.1), described in 2009 in China, ALV-E B10 (KC610516.1) in 2013 in Canada, and ALV-E TYR (MT263508.1) and ros008 (MT263515), both detected in 2020 in the United Kingdom. These were grouped together in 91 % of the replicated trees, with a potential common ancestral to Crax 1 and these strains. Except for Crax 1, all compared strains were found in chickens (*Gallus gallus domesticus*), and described worldwide. Chicken is a major animal production industry in Brazil and worldwide, and the potential cross transference of pathogens exists. In addition, free-range poultry is commonplace in Brazil and elsewhere, and known to harbor relevant pathogens, such as viruses [27, 28]. On the tree, at the precursor branches, the strains ev-C11 (DQ500007) of South Korea, described in 2008, CR-1986 (GU002400), described in 1986 in Costa Rica, and EAV-HP (AJ238125), described in 2005 in the UK, seemed to be related to a common ancestral to all strains in the tree, including Crax 1. CR-1986 was described in Costa Rica associated to novel ALV associated with osteopetrosis. However, the phylogenetic relationships could not be associated to a chronological or geographical parameter.

## Discussion and conclusions

To the best of our knowledge, this is the first report of an osteosarcoma in a bare-faced curassow with an unusual polyostotic manifestation and associated with ALV infection. Polyostotic presentation of osteosarcoma was previously described in a wild kestrel (*Falco tinnunculus*), affecting joints of the left limb (knee and tarsus) with similar lesions in the tarsometatarsus of the contralateral limb [29]. However, in that case there were no investigation of virus infection and no detectable metastasis [29]. Neoplasms were investigated in raptor birds in the UK and revealed the occurrence of osteosarcoma in a captive gyr/saber falcon hybrid (*Falco rusticolus*/*Falco cherrug*) and in a free-living Eurasian buzzard (*Buteo buteo*) [30]. Although rare, polyostotic presentation was also described in a domestic dog, which had multiples sites of neoplastic growth in the appendicular skeleton and also had kidney metastasis [31]. In domestic animals, osteosarcoma usually results in metastasis, which frequently affects the lungs, kidney, and liver [4–6].

ALV infection is associated with the development of various types of mesenchymal neoplasms, including myxosarcomas, hemangiosarcomas, leiomyosarcomas, neurofibromas, and hemagiomas [32–35]. Importantly, osteosarcoma has not been previously reported in a Cracid infected with ALV, thus expanding our knowledge on host range and pathogenesis potential of this virus. Osteopetrosis, on the other hand, is a common proliferative lesion in chickens infected with ALV, and usually have a multiple sites presentation, with involvement of both limbs [19]. Interestingly, the pattern of growth of the excessive osseous matrix in osteopetrosis is from the cortex, extending internally, filling all pneumatic or medullar space, and externally, leading to the compression of nerves and adjacent muscle tissue. That was the growth pattern observed in this case, in which the cells were pleomorphic, with malignant features, and resulted in renal metastasis. Previous studies demonstrated that ALV is capable of changing the differentiation and growth activity of osteoblasts [19], and considering the vastly documented oncogenic potential of this virus, may support the notion of a retroviral malignant neoplastic proliferation arising from an initial osteopetrotic lesion.

Bare-faced curassow is considered a vulnerable species so the infection with ALV may have an impact on conservation programs, since many animals may be asymptomatic with the possibility of vertical transmission. In some situations this virus may be responsible for outbreaks of neoplasms that may have a lethal evolution [32, 33]. Finally, this report highlights the importance of ALV in association with a malignant and metastatic neoplasm and the need of active and constant surveillance, especially in threatened wild gallinaceous species.

## Data Availability

All relevant data are within this paper. The datasets generated during the current case study are available from the corresponding author on reasonable request. DNA sequencing data generated in this study is available at https://www.ncbi.nlm.nih.gov/nucleotide/, under accession number MN553590.1.

## Abbreviations

ALV: Avian leukosis virus
DNA: Deoxyribonucleic acid
HE: Hematoxylin and eosin
PCR: Polymerase chain reaction
REV: Reticuloendotheliosis virus
UK: United Kingdom
